# In vitro and in vivo effects of flubendiamide and copper on cyto-genotoxicity, oxidative stress and spleen histology of rats and its modulation by resveratrol, catechin, curcumin and α-tocopherol

**DOI:** 10.1186/s40360-020-00405-6

**Published:** 2020-04-23

**Authors:** Rajesh Mandil, Atul Prakash, Anu Rahal, S. P. Singh, Deepak Sharma, Rahul Kumar, Satish Kumar Garg

**Affiliations:** 1 Department of Veterinary Pharmacology and Toxicology, College of Veterinary and Animal Sciences, Sardar Vallabhbhai Patel University of Agriculture and Tecahnology, 250110, Meerut, India; 2 Department of Veterinary Pharmacology and Toxicology, College of Veterinary Science and Animal Husbandry, U.P. Pt. Deen Dayal Upadhyay Pashu Chikitsa Vigyan Vishwavidyalaya Evam Go- Anusandhan Sansthan (DUVASU), -281001, Mathura, India; 3 Division of Goat Health, Central Institute for Research on Goat (CIRG), Makhdoom, Farah, Mathura, Uttar Pradesh 281122 India; 4 Department of Animal Genetics & Breeding, College of Veterinary Science and Animal Husbandry, U.P. Pt. Deen Dayal Upadhyay Pashu Chikitsa Vigyan Vishwavidyalaya Evam Go-Anusandhan Sansthan (DUVASU), 281001, Mathura, India; 5 Department of Veterinary Pathology, College of Veterinary Science and Animal Husbandry, U.P. Pt. Deen Dayal Upadhyay Pashu Chikitsa Vigyan Vishwavidyalaya Evam Go-Anusandhan Sansthan (DUVASU), 281001, Mathura, India

**Keywords:** Flubendiamide, Copper, Splenocytes, Cyto-genotoxicity, Oxidative stress

## Abstract

**Background:**

Living organisms are frequently exposed to more than one xenobiotic at a time either by ingestion of contaminated food/fodder or due to house-hold practices, occupational hazards or through environment. These xenobiotics interact individually or in combination with biological systems and act as carcinogen or produce other toxic effects including reproductive and degenerative diseases. Present study was aimed to investigate the cyto-genotoxic effects of flubendiamide and copper and ameliorative potential of certain natural phyotconstituent antioxidants.

**Method:**

In vitro cytogenotoxic effects were evaluated by employing battery of assays including Propidium iodide staining, Tunel assay, Micronuclei, DNA fragmentation and Comet assay on isolated splenocytes and their prevention by resveratrol (5 and 10 μM), catechin (10 and 20 μM), curcumin (5 and 10 μM) and α-tocopherol (5, 10 and 20 μM). In vivo study was also undertaken daily oral administration of flubendiamide (200 mg/kg) or copper (33 mg/kg) and both these in combination, and also all these concurrently with of α-tocopherol to Wistar rats for 90 days.

**Results:**

Flubendiamide and copper produced concentration-dependent cytotoxic effects on splenocytes and at median lethal concentrations, flubendiamide (40 μM) and copper (40 μM) respectively produced 71 and 81% nonviable cells, higher number of Tunel+ve apoptotic cells, 7.86 and 9.16% micronucleus and 22.90 and 29.59 comets/100 cells and DNA fragmentation. In vivo study revealed significant (*P* < 0.05) increase in level of lipid peroxidation (LPO) and decrease in glutathione peroxidase (GPx), glutathione-S-transferase (GST) and superoxide dismutase (SOD) activities in groups exposed to flubendiamide or copper alone or both these in combination. Histopathological examination of rat spleens revealed depletion of lymphoid tissue, separation of splenocytes and rarification in splenic parenchyma of xenobiotic(s) treated groups.

**Conclusion:**

Flubendiamide and copper induce oxidative stress and produce cytogenotoxic effects along with histoarchitectural changes in spleen. All four tested natural antioxidants (resveratrol, catechin, curcumin and α-tocopherol) reduced flubendiamide and copper-induced cytotoxic effects in rat splenocytes. Rat splenocytes are very sensitive to flubendiamide and copper-induced cytogenotoxicity, therefore, these can be effectively employed for screening of compounds for their cytogenotoxic potential. α-tocopherol was effective in restoring alterations in oxidative stress biomarkers and preventing histoarchitectural lesions in spleen.

## Background

Last few decades toxicological research has revealed that immune system is the potential target for xenobiotics-induced adverse effects due to exposure to environmental pollutants, indiscriminate use of agrochemicals, metals, drugs, other chemicals and their metabolites. Therefore, the present study was undertaken to investigate the cyto-genotoxic potential of flubendiamide and copper in rat splenocytes primary cell culture following in vitro exposure. In vivo effect of these xenobiotics on oxidative stress biomarkers and histopathological changes in rat spleen were also studied. Ameliorative potential of α-tocopherol and other plants-based antioxidants against the adverse effects of these xenobiotics was also evaluated. For in vivo study, Wistar rats were orally exposed to flubendiamide or copper alone, both these in combination, and also along with α-tocopherol for 90 days.

Flubendiamide is a comparatively new insecticide and selectively acts on insects ryanodine receptors (RyR). It possesses favourable toxicological profile due to its higher (> 2000 mg/kg) oral and dermal LD^50^ values in rats. Being comparatively safe, it is being widely used on large number of crops which include fruits, vegetable crops and nuts to control insects. Therefore, human beings and animals are also being indiscriminately exposed to flubendiamide through direct and indirect routes. Genotoxicity is the primary risk factor associated with long-term exposure to environmental pollutants including insecticides and metals. Flubendiamide does not have genotoxic effects on bone marrow cells [1–6]. But there are reports that exposure to certain xenobiotics, either individually or in combination, may result in gene mutation, chromosomal aberrations and DNA damage [7–9].

Copper, being a micronutrient, is essential for life of humans and animals and is required in minute concentrations for functioning of several metalloenzymes [10–12]. It also possesses fungicidal, molluscicidal and weedicidal activities and is employed for control of bacterial and fungal diseases of fruits, vegetables, nuts and field crops, algae in domestic lakes and ponds and in gardening as powder and spray [13, 14]. In India, copper also enters in human body through drinking water, and inhalation of copper dust and fumes [15]. But it is toxic when present in the body in excess [10].

Environmental pollutants increase oxidative stress [16] and dietary antioxidants prevent free radicals induced tissue damage by preventing formation of radicals, scavenging them, or by promoting their decomposition [17–19]. Several natural food-derived components have received great attention in recent years as nutraceuticals due to their promising biological activities. α-tocopherol (α-TOH) is the major lipid soluble natural form of vitamin E and possesses antioxidant property. It protects cellular membrane and lipoproteins from peroxidation by reacting with lipid radicals produced in lipid peroxidation chain reaction [20–22]. Green tea is very rich in phenolic compounds including catechin and epigallocatechin gallate (EGCG) [23]. These are powerful antioxidant, inhibit apoptosis by inhibiting caspase 3 activity thereby preventing expression of proapoptotic (Bax, Bad and Mdm2) and antiapoptotic genes (Bcl-2, Bcl-w and Bcl-xL) to protect SH-SY5Y cells from 6-OHDA-induced apoptosis [24–26] and EGCG is cancer chemopreventive also [27]. Curcumin is the main coloring agent of turmeric, used as a spice in India, and possesses number of promising pharmacological activities including antioxidant [28–31] and DNA protective effect against arsenic, fluoride and chlorpyriphos [32–34]. Phytoallexin resveratrol, found in the skin of grapes, possesses the potential to inhibit cancer initiation, promotion and progression, and inhibits TNFα-induced reactive oxygen intermediate generation [35–37].

In view of the sparse information on in vitro cyto-genotoxicity potential and in vivo adverse effects of flubendiamide in mammals, and conflicting reports on genotoxic effects of copper, the present study was undertaken. We also evaluated the ameliorative potential of certain natural phyotconstituent antioxidants against these xenobiotics to explore their therapeutic and prophylactic use.

## Methods

### Experimental animals and chemicals

Present study was undertaken on Wistar rats, which were procured from Laboratory Animal Resource Section, Indian Veterinary Research Institute, Izatnagar, India and maintained under standard managemental conditions in the Departmental Experimental Animal House. Animals had free access to pelleted feed (Ashirwad Industries, Chandigarh) and clean and deionized drinking water. Daily light and dark cycle of 12 h was ensured. Before start of the experiment, an acclimatization period of 15 days was allowed. Whole study was carried out in two phases: Phase I - in vitro apoptosis studies while Phase II included only in vivo studies.

The study was approved by the Institutional Animal Ethics Committee (IAEC; 79 IAEC/13). Flubendiamide, dexamethasone, resveratrol, catechin, curcumin, and α-tocopherol were procured from Sigma-Aldrich (USA) while copper sulphate from Sd Fine Chemical Ltd.

### Phase I- in vitro study

Twenty adult male Wistar rats weighing 80–100 g were used for in vitro cyto-genotoxicity study on primary cell culture of isolated rat splenocytes.

### Isolation of splenocytes

Rats were sacrificed by cervical dislocation and spleen was aseptically removed and quickly disintegrated into many pieces. Vigorous pipetting of meshed tissue was done with the help of 10 ml glass pipette to break the minced tissue and these cells were transferred to 15 ml test tubes containing chilled PBS and allowed to stand on ice for 15 min. Top 12 ml of suspension was collected into another centrifuge tube and cells were pelleted by centrifugation at 1500 rpm for 10 min. Cells pellet was re-suspended in PBS and centrifuged again at 1500 rpm for 10 min. The supernatant was discarded and pellet was treated with 5 ml of RBC lysis buffer (4.15 g NH_4_Cl; 0.5 g NaHCO_3_; 0.0186 g Na_2_-EDTA; 200 ml DW) and kept for 10 min in ice and centrifuged at 1500 rpm for 10 min. Then the pellet was given two washings with PBS at 1500 rpm for 10 min. The pellet was re-suspended in 1 ml of Roswell Park Memorial Institute (RPMI-1640; Sigma-Aldrich) medium with 10% foetal calf serum (Sigma-Aldrich). Viability count was done using 0.1% trypan blue exclusion test and the cells density was adjusted to obtain 10^6^ cells/ml [38].

### Median lethal concentrations

Isolated splenocytes were seeded in 24 well culture plates containing 10^6^ cells/ml in 10% RPMI with foetal calf serum. Different concentrations of flubendiamide and copper i.e. 1.0, 2.5, 5, 7.5, 10, 15, 20, 40, 60, 80 and 100 μM were used. Culture plates were incubated for 12 h in CO_2_ incubator (New Brunswick Scientific, USA) at 37 °C with 5% CO_2._ After incubation, samples were collected in 1.5 ml eppendorf tubes and centrifuged at 3200 rpm for 10 min. Supernatant was discarded and the pellet was dissolved in 0.5 ml PBS. Propidium iodide (Sigma) was added at 1 μg/ml concentration to cells and incubated for another 15 min in dark at room temperature. Cells were observed under fluorescent microscope (Microscan 20 PFM, Nitco) under green filter to determine the approximate concentrations of test xenobiotics at which almost 50% dead splenocytes were observed. Calculation of the LC50 value of flubendiamide and copper was done by subjecting the data (concentrations used versus % cell dead) of Table 1 to “Probit Analysis method” using “Graph Pad Prism software” and by plotting the log values of the concentrations of xenobiotics used against log values of the per cent cells dead. Further we interpolated the respective log values of the xenobiotics (copper and flubendiamide) at which 50% of the cells are expected to be dead, and then the antilog values of these log values were calculated. It is apparent that the interpolated LC50 value for copper was 38.90 μM and for flubendiamide, it was 37.23 μM. Both these values are very close to 40 μM and considered as median lethal concentration of flubendiamide and copper and used for further studies.

**Table 1:** Effect of different concentrations of flubendiamide and copper on per cent viability of rat splenocytes following their in vitro exposure to these xenobiotics

Treatment	% Dead Splenocytes
Control	4.93 ± 0.67
Vehicle control (DMSO) 50 μl	7.46 ± 0.83
1.0 μM Flubendiamide	12.98 ± 1.92
2.5 μM Flubendiamide	15.13 ± 1.87
5.0 μM Flubendiamide	22.18 ± 1.67
7.5 μM Flubendiamide	24.28 ± 2.24
10 μM Flubendiamide	28.09 ± 1.33
15 μM Flubendiamide	29.76 ± 1.55
20 μM Flubendiamide	32.32 ± 1.32
40 μM Flubendiamide	45.42 ± 2.50
60 μM Flubendiamide	67.89 ± 3.14
80 μM Flubendiamide	88.81 ± 5.62
1.0 μM Copper	6.45 ± 3.04
2.5 μM Copper	10.34 ± 1.63
5.0 μM Copper	13.88 ± 1.39
7.5 μM Copper	16.66 ± 1.92
10 μM Copper	26.08 ± 4.73
15 μM Copper	28.39 ± 1.74
20 μM Copper	34.95 ± 5.87
40 μM Copper	51.09 ± 2.01
60 μM Copper	61.11 ± 2.03
80 μM Copper	76.68 ± 1.71

Data presented are Mean ± SEM of three observations

### Viability of splenocytes

Freshly collected splenocytes (10^6^ cells/ml) were exposed to median lethal concentrations of flubendiamide and copper alone, and also along with the antioxidants- resveratrol (5 and 10 μM), curcumin (5 and 10 μM), catechin (10 and 20 μM) and α-tocopherol (5, 10 and 20 μM). Solutions of resveratrol, catechin, curcumin, α-tocopherol, flubendiamide, copper sulphate and dexamethasone were prepared in dimethyl sulphoxide (DMSO). Culture plates were incubated for 12 h in CO_2_ incubator at 37 °C with 5% CO_2_ and further processed as described above to determine the number of nonviable cells.

### TUNEL assay

After exposure of splenocytes to median lethal concentrations of flubendiamide (40 μM) and copper (40 μM) for 12 h, these samples were further processed for determination of apoptosis as per the protocol described in TUNEL Assay Kit (Invitrogen, USA; Ref. No. A35126). Apoptotic cells, which underwent extensive DNA degradation during late stages of apoptosis, were examined under blue filter of fluorescent microscope. Cells which fluoresced brightly were considered as apoptotic.

### Genotoxicity assays (micronucleus, DNA fragmentation and comet)

#### Micronucleus assay

Flubendiamide and copper genotoxicity potential was assessed by micronuclei assay by using the isolated splenocytes [39]. 10^6^ cells/ml were incubated with flubendiamide (40 μM) and copper (40 μM) alone and with different μM concentrations of resveratrol, catechin, curcumin and α-tocopherol and incubated for 12 h in CO_2_ incubator. After incubation, samples were collected in 1.5 ml eppendorf tubes and centrifuged at 3200 rpm for 10 min. Supernatants were discarded and the pellets were dissolved in 1.0 ml of Hank’s balanced salt solution (HBSS) having pH 7.2 and centrifuged again for 10 min at 3200 rpm. Supernatant was removed and cells in suspension were mixed carefully in 100 μl of HBSS. A drop of cell suspension was taken on grease-free clean glass slide and smeared. The smear was air-dried and fixed with absolute methanol (100%) for 5 min and stained with acridine orange for 1 min at room temperature. The slide was rinsed in Sorensen’s buffer (pH 6.8) and kept for at least 3 min and this step was repeated three times. Slides were examined on the same day and 1000 cells (both mononuclear and binucleated) per slide were scored under green filter of the fluorescent microscope to determine the frequency of micronuclei formation.

#### DNA fragmentation assay

DNA ladder assay was performed according to phenol-chloroform-DNA isolation protocol [40]. After incubation of 5 X10^6^ cells each with flubendiamide or copper alone and with antioxidants, as mentioned in micronuclei assay method, the cells were collected in 1.5 ml of eppendorf tubes and centrifuged at 3200 rpm for 10 min at 4 °C. The cells pellet was washed with PBS having pH 7.2, mixed with DNA extraction buffer (500 μl/tube) and kept in water bath for 1.0 h at 37 °C. 10% SDS was added (20 μl/ml) to the cell suspension and tubes were gently mixed by inverting the tubes. Contents of the tubes appearing viscous indicated lysis of splenocytes. Proteinase K (15 μl of 20 mg Proteinase K/ml of buffer) was added to each tube in two pulses i.e. half the requirement was added to tube in the 1st pulse and mixed gently and kept in water bath at 50 °C. After 3–4 h, a second pulse of the remaining amount of proteinase K was added. Tubes were incubated at 50 °C overnight. Next day morning, equal amount of equilibrated phenol (Tris saturated phenol pH > 7.8) was added to each tube and mixed by gently inverting the tubes for 15 min till light coffee coloured uniform solution was formed and centrifuged at 3400 rpm for 15 min. The upper aqueous phase containing DNA was transferred into fresh 1.5 ml clean eppendorf tube. Similar extraction was done (as in the above step) once with equal volume of phenol: chloroform: isoamyl alcohol (25:24:1) and with chloroform: isoamyl alcohol (24:1). To obtain the final aqueous phase, double the volume of chilled (− 20 °C) ethanol was added. Tubes were mixed gently by inversion and kept at room temperature to allow precipitation of DNA. DNA pellet was washed twice with 500 μl of 70% ethanol and eppendorf tube was centrifuged at 10000 rpm for 10 min at room temperature. Finally 70% ethanol was discarded and DNA pellet was air dried by inverting tube on blotting paper so that last traces of ethanol were removed. However, it was ensured that pellet did not over-dry so to enable an easy dissolution in the following step. Approximately 50 μl of tris-EDTA buffer (TE) was added and kept in water bath at 60 °C for 2 h to inactivate DNAse and other enzymes. Eppendorf was stored at 4 °C for a week so that DNA was dissolved. DNA concentration and its purity was determined spectrophotmetrically by Biophotometer plus (Eppendorf) at 260 and 280 OD. Integrity of the DNA was examined in agarose gel (1.0%) electrophoresis and visualized under UV light in gel documentation system after staining with ethidium bromide.

#### Comet assay

Single splenocyte cells were isolated from spleen after cervical dislocation and viability checked by Trypan blue exclusion test. 5X10^6^ cells/well were kept for culturing and treated with flubendiamide and copper (40 μM/well) alone and with different micromolar concentrations of resveratrol, catechin, curcumin and α-tocopherol. After incubation of 12 h in CO_2_ incubator, cells were collected in 1.5 ml eppendorf tubes and centrifuged at 3200 rpm for 10 min at 4 °C. Supernatant was discarded and the pellet was washed with PBS (pH 7.2). Comet assay was performed using the standard method with normal (NMA) and low melting agarsoe (LMPA) [41].

Briefly, slides were dipped in methanol and heated over blue flame to remove the grease, dust and oil. 1.5% NMA (Sigma-Aldrich) and 0.5% LMPA (Sigma–Aldrich) were prepared in PBS. LMP agarose was kept in water bath at 40 °C to cool and stabilize while NMA agarose was kept at 100 °C. First layer of agarose on the slides was prepared by dipping conventional pre-cleaned slide for few seconds in 100 ml wide mouth beaker containing 1.5% NMA up to one-third area and gently removed. Underside of the slide was wiped to remove excess agarose and allowed to dry in a tray. Slides were generally prepared a day earlier. Splenocyte cell pellets were uniformly mixed with 100 μl of 0.5% LMPA and poured carefully on the first agarose layer and immediately covered with a full length cover slip. Slides were kept on ice-pack for 15–20 min to allow for the 2nd agarose layer to solidify. After solidification, the cover slip was removed and the slide was kept in a coupling jar containing freshly prepared lysis solution (1 ml-Triton X-100 and 10 ml DMSO was added to 89 ml stock lysing solution containing NaCl-36.52 g; EDTA disodium salt-9.3 g; Trizma-0.3 g; NaOH-2 g- For 250 ml) at 4 °C overnight. Next day, the slide was removed from lysis solution and kept for 30 min in freshly prepared electrophoretic buffer so as to cause unwinding of DNA and expression of alkali-labile sites. Slide was run in horizontal electrophoresis (Bio Rad) chamber with the same electrophoresis buffer (pH > 13) at 25 V and 300 mA for 1 h. After running in electrophoresis chamber, the slide was gently removed and placed horizontally in a tray and covered with neutralizing buffer for 5 min and then decanted it; the same step was repeated three times to remove alkali and detergent. This step was critical to bring down the pH from 13 to 7.5. After neutralization, slides were stained by placing 3–4 drops of 100 μl working ethidium bromide solution at equal distance and immediately covered with cover slip. Slides were examined under fluorescent microscope, individual cell/comets were observed and images were captured at 40X magnification using green filter and duplicate slides per treatment were observed. At least 50 cells from each slide were scored and a total of 100 cells/treatment was scored to get the reproducible data.

#### Phase II-in vivo chronic toxicity study

Fifty four adult male Wistar rats weighing between 130 and 150 g were divided in nine groups of six animals each. Animals of six groups (IV to IX) were orally treated on daily basis with copper (33 mg/kg; group IV), flubendiamide (200 mg/kg; group V) or combination of both these (group VI), and α-tocopherol (100 mg/kg) along with these xenobiotics singly (group VII and VIII) or both these in combination (group IX) for 90 days. Groups I and II served as negative and vehicle controls (corn oil), respectively while rats of group III were administered only α-tocopherol (100 mg/kg). Solutions of copper sulphate and flubendiamide (FAME®, Bayer) were prepared in deionized water while α-tocopherol was dissolved in corn oil. Doses of flubendiamide and copper were 1/10th of the LD_50._ At the end of exposure period, rats were humanely sacrificed by cervical dislocation and their spleen was collected and blotted with tissue paper. It was then used to determine its levels of different oxidative stress related parameters such as lipid peroxidation (LPO), reduced glutathione (GSH), catalase (CAT), superoxide dismutase (SOD), glutathione-S-transferase (GST) and glutathione peroxidase (GPx), along with total protein content in splenic tissue using UV- VIS spectrophotometeric methods [42–48]. 200 mg of the spleen sample was weighed and transferred in 2 ml of chilled saline. The same weight of the spleen sample was separately taken in 2 ml of 0.02 M EDTA for GSH estimation. Tissue homogenates were prepared by using tissue homogenizer (Heidolph) under cold conditions and centrifuged for 10 min at 3000 rpm. The supernatant was used for estimation of different oxidative stress biomarkers. Lipid peroxidation (LPO) and reduced glutathione (GSH) were assayed immediately after tissue collection.

A small piece of the spleen tissue was collected in 10% formaldehyde saline solution and processed for preparation of paraffin blocks as per the method described by [49]. Tissue sections of 5–6 μm thickness were cut using a microtome (Leica, Germany) and stained with haematoxylin and eosin. Microscopic slides were examined under light microscope to observe the histoarchitecture changes in spleen.

### Statistical analysis of data

Data of the in vitro study has been presented as Mean ± SEM of the three observations in each treatment group in Tables 1 and 2. Table 3 presents the Mean ± SEM data of in vivo study. Effects of different in vitro treatments were compared between the control and xenobiotics alone-treated groups, and also between the xenobiotics alone and those treated concurrently with antioxidants. Statistically significant differences between the different treatment groups observed in in vivo study were determined using one-way ANOVA followed by Tukey’s multiple post-hoc test with the help of SPSS® 16 software. Significant difference was considered at *P* < 0.05.

**Table 2:** Effect of median lethal concentrations of flubendiamide and copper alone and in the presence of different concentrations of resveratrol, catechin, curcumin and α-tocopherol on viability, micronuclei and comet formation in rat splenocytes following their in vitro exposure

Treatments	^**a**^Nonviable cells (%)	^**a**^Micronuclei (%)	No. of Comet/ 100 cells (%)
Control	5.41 ± 0.33	0.96 ± 0.08	3.09 ± 0.31
DMSO (50 μl)	8.59 ± 0.88	1.36 ± 0.08	4.58 ± 0.28
Dexamethasone (20 μM)	–	7.60 ± 0.20	27.69 ± 0.87
Flubendiamide (40 μM)	71.88 ± 2.90	7.86 ± 0.17	22.90 ± 0.90
Resveratrol (5 μM) + Flubendiamide (40 μM)	50.00 ± 1.85	1.20 ± 0.17	20.15 ± 1.91
Resveratrol (10 μM) + Flubendiamide (40 μM)	24.36 ± 0.88	1.10 ± 0.05	15.44 ± 1.47
Catechin (10 μM) + Flubendiamide (40 μM)	53.66 ± 1.76	1.43 ± 0.24	14.80 ± 1.25
Catechin (20 μM) + Flubendiamide (40 μM)	52.46 ± 2.33	1.33 ± 0.08	12.64 ± 0.57
Curcumin (5 μM) + Flubendiamide (40 μM)	56.25 ± 3.05	3.13 ± 0.12	7.58 ± 0.89
Curcumin (10 μM) + Flubendiamide (40 μM)	38.24 ± 3.18	1.40 ± 0.15	7.20 ± 0.32
α-tocopherol (5 μM) + Flubendiamide (40 μM)	55.00 ± 0.33	2.10 ± 0.40	11.56 ± 0.33
α-tocopherol (10 μM) + Flubendiamide (40 μM)	40.26 ± 2.02	1.93 ± 0.29	6.96 ± 0.30
α-tocopherol (20 μM) + Flubendiamide (40 μM)	17.65 ± 0.57	3.30 ± 0.26	4.89 ± 0.33
Copper (40 μM)	81.11 ± 6.06	9.16 ± 0.21	29.59 ± 1.76
Resveratrol (5 μM) + Copper (40 μM)	76.54 ± 4.84	4.80 ± 0.20	25.33 ± 0.47
Resveratrol (10 μM) + Copper (40 μM)	30.43 ± 4.04	1.90 ± 0.32	9.69 ± 0.66
Catechin (10 μM) + Copper (40 μM)	72.13 ± 3.71	5.20 ± 0.20	15.12 ± 0.32
Catechin (20 μM) + Copper (40 μM)	65.82 ± 1.41	2.10 ± 0.36	12.41 ± 1.20
Curcumin (5 μM) + Copper (40 μM)	64.18 ± 3.84	3.47 ± 0.14	16.80 ± 0.87
Curcumin (10 μM) + Copper (40 μM)	59.68 ± 4.33	2.63 ± 0.21	12.71 ± 1.32
α-tocopherol (5 μM) + Copper (40 μM)	59.72 ± 5.0	3.33 ± 0.24	10.33 ± 1.20
α-tocopherol(10 μM) + Copper (40 μM)	54.73 ± 4.91	1.93 ± 0.18	15.20 ± 1.45
α- tocopherol (20 μM) + Copper (40 μM)	51.06 ± 4.18	3.16 ± 0.26	10.61 ± 0.66

^a^Data presented are Mean + SEM of three observations

**Table 3:** Effect of oral administration of α-tocopherol (100 mg/kg) on certain splenic oxidative stress biomarkers following 90 days oral exposure of rats to copper (33 mg/kg) and flubendiamide (200 mg/kg) alone and both in combination (33 mg/kg + flubendiamide 200 mg/kg)

Groups	Treatment	Protein in splenic tissue (mg/ml)	LPO (nM MDA/g tissue)	SOD^**a**^ (U/ mg of protein)	Catalase (mM H_2_O_2_ utilized/min mg of protein)	GSH (mM GSH/g tissue)	GST^**a**^(μM of CDNB-GSH conjugate min-^1^ mg-^1^ protein)	GPx (nM NADPH utilized/min/mg protein)
**I**	Control	3.42 ± 0.56 ^a^	50.56 ± 1.60^a^	3.93 ± 0.35^a^	85.32 ± 7.35^a^	2.38 ± 0.21^abc^	1.62 ± 0.12^a^	1.93 ± 0.35^a^
**II**	Vehicle control (Corn oil)	4.05 ± 1.02 ^a^	61.78 ± 2.12^ab^	4.16 ± 0..36^a^	79.07 ± 10.61^a^	2.48 ± 0.17^ab^	1.74 ± 0.14^a^	0.96 ± 0.37^ab^
**III**	α-tocopherol (100 mg/kg)	3.21 ± 0.41 ^a^	68.56 ± 8.08^ab^	3.33 ± 0.40^ab^	87.94 ± 9.15^a^	2.49 ± 0.08^a^	1.66 ± 0.15^a^	0.59 ± 0.06^ab^
**IV**	Copper sulphate (33 mg/kg)	2.59 ± 0.32 ^a^	81.65 ± 4.08^b^	1.82 ± 0.25^b^	80.54 ± 7.29^a^	1.91 ± 0.04^abc^	1.65 ± 0.20^a^	0.49 ± 0.04^b^
**V**	Flubendiamide (200 mg/kg)	3.69 ± 0.61 ^a^	81.70 ± 5.57^b^	3.40 ± 0.38^a^	60.37 ± 7.99^a^	1.84 ± 0.00^c^	0.61 ± 0.12^b^	0.37 ± 0.06 ^b^
**VI**	Copper sulphate (33 mg/kg) + Flubendiamide (200 mg/kg)	2.30 ± 0.34 ^a^	75.06 ± 7.67^b^	3.27 ± 0.34^ab^	98.76 ± 10.80^a^	1.96 ± 0.12^abc^	1.34 ± 0.25^a^	0.43 ± 0.11^b^
**VII**	Copper sulphate (33 mg/kg) + α-tocopherol (100 mg/kg)	2.81 ± 0.37 ^a^	77.13 ± 10.74^b^	3.02 ± 0.25^ab^	83.48 ± 8.79^a^	1.87 ± 0.09^bc^	1.50 ± 0.09^a^	1.16 ± 0.45^ab^
**VIII**	Flubendiamide (200 mg/kg) + α-tocopherol (100 mg/kg)	4.16 ± 0.52 ^a^	61.14 ± 6.50^ab^	4.42 ± 0.40^a^	61.74 ± 10.55^a^	2.19 ± 0.19^abc^	1.43 ± 0.15^a^	0.68 ± 0.36^ab^
**IX**	Copper sulphate (33 mg/kg) + Flubendiamide (200 mg/kg)) + α-tocopherol (100 mg/kg)	2.85 ± 0.27 ^a^	68.96 ± 4.84^ab^	3.05 ± 0.37^ab^	81.40 ± 7.19^a^	2.24 ± 0.10^abc^	1. 95 ± 0.30^a^	1.09 ± 0.48^ab^

• Values (Mean ± SEM; *n* = 6) bearing different superscripts in the same column differed significantly (*P* < 0.05)

• ^a^Values are Mean ± SEM of five animals only

## Results

### Phase I- in vitro study

#### Median lethal concentrations

Data on in vitro effect of different concentrations of flubendiamide (1.0–80 μM) and copper (1.0–80 μM) on rats splenocytes revealed concentration-dependent lethal effect of these xenobiotics. There was dose-dependent increase in percentage of the nonviable splenocytes and nearly 50 % nonviable splenocytes were observed between 40 μM and 60 μM concentrations of these xenobiotics (Table 1). Therefore, 40 μM was considered the approximate median lethal concentration both for flubendiamide and copper.

### Viability of splenocytes

Fluorescent microscopic examination of flubendiamide (40 μM) and copper (40 μM) alone-treated splenocytes respectively showed 71.88 and 81.11% nonviable cells compared to 5.41% in control and 8.59% in DMSO-treated cells (Table 2). Following concomitant in vitro treatment of splenocytes with xenobiotics and antioxidants- resveratrol, catechin, curcumin and α-tocopherol, the percentage of the nonviable splenocytes was found to decrease and effect of all these four antioxidants was concentration-dependent (Table 2). Out of these tested antioxidants, based on their comparative efficacy on equi-molar concentration basis (10 μM), resveratrol was found to be the most effective against flubendiamide in reducing the percentage of nonviable splenocytes, and the order of ameliorative potential of these antioxidants was: resveratrol > curcumin ≈ α-tocopherol > catechin (Table 2). Similarly, resveratrol was also found to be the most effective against copper-induced viability losses in splenocytes; and the order of ameliorative potential against copper was: resveratrol > α-tocopherol > curcumin > catechin.

### Tunel assay

**Fig. 1:**
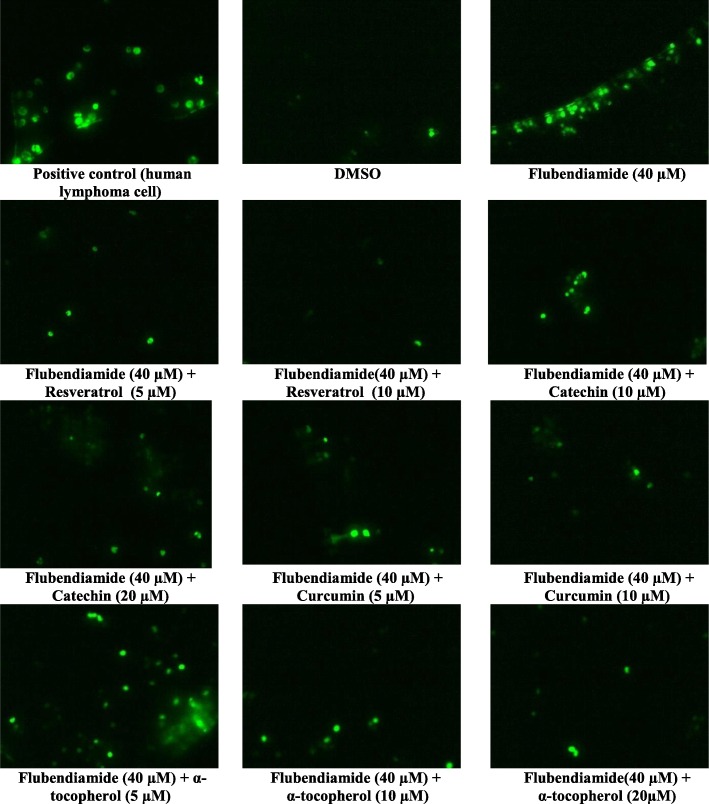
Representative photographs of rat splenocytes showing TUNEL + ve cells (40 X) following in vitro exposure to median lethal concentration of flubendiamide alone (40 μM) and in the presence of different concentrations of natural antioxidants-resveratrol, catechin, curcumin and α-tocopherol

**Fig. 2:**
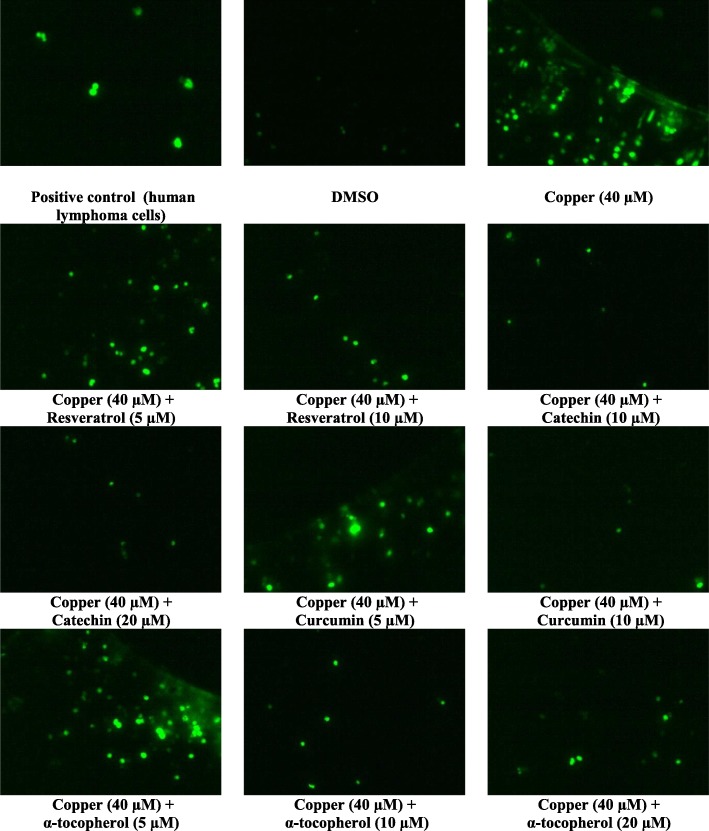
Representative photographs of rat splenocytes showing TUNEL + ve cells (40 X) following in vitro exposure to median lethal concentration of copper alone (40 μM) and in the presence of different concentrations of natural antioxidants-resveratrol, catechin, curcumin and α-tocopherol

Splenocytes exposed to 40 μM flubendiamide or copper showed higher number of Tunel-positive (Tunel+ve) cells compared to those in negative or vehicle control (DMSO) groups as shown in Figs. 1 and 2, respectively. Compared to flubendiamide, copper was more potent in producing Tunel+ve splenocytes, and compared to the flubendiamide or copper-alone treated splenocytes, marked reduction in Tunel+ve cells was observed in the splenocytes treated concurrently with either of the xenobiotic (flubendiamide or copper) and different antioxidants (resveratrol 5 and 10 μM, catechin 10 and 20 μM, curcumin 5 and 10 μM or α-tocopherol 5, 10 and 20 μM) as shown in Figs. 1 and 2. However, based on the efficacy of different antioxidants at equimolar concentration basis i.e. 10 μM, resveratrol was most effective in reducing the number of Tunel+ve cells induced by flubendiamide (Fig. 1) and the overall order of efficacy of different antioxidants was resveratrol > curcumin >α-tocopherol > catechin. Just like their efficacy against flubendiamide, all these were effective in reducing copper-induced increase in number of Tunel+ve cells and the overall order of efficacy of different antioxidants was curcumin > catechin ≥ α-tocopherol ≥ resveratrol (Fig. 2). However, contrary to resveratrol, curcumin was most effective against copper.

### Micronuclei formation

**Fig. 3:**
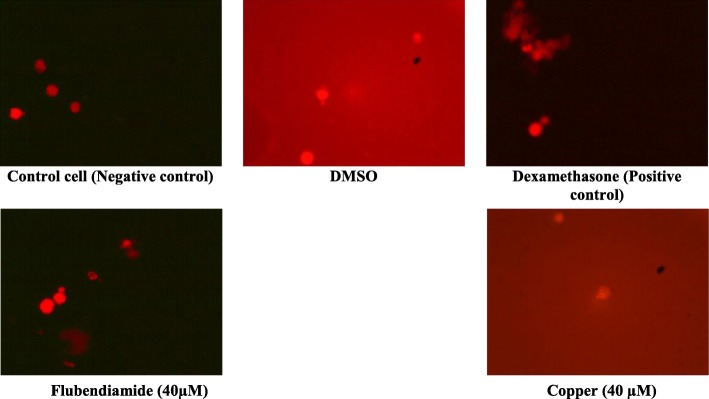
Representative photographs of rat splenocytes showing micronuclei formation (100 X) following in vitro exposure to median lethal concentrations of flubendiamide and copper alone (40 μM) and in the presence of dimethyl sulphoxide (DMSO) and dexamethasone

Flubendiamide and copper alone treated splenocytes showed micronuclei formation in 7.86 and 9.16**%** cells respectively compared to 0.96% in negative control and 1.36% in DMSO-treated splenocytes (Table 2; Fig. 3). Dexamethasone-induced micronuclei formation (7.6%) was much higher compared to that in negative control and DMSO-treated splenocytes. Almost a similar percentage of micronuclei were observed in splenocytes treated with flubendiamide (7.86%) or copper (9.16%) as summarized in Table 2. Ameliorative efficacy studies with resveratrol, catechin, curcumin and α-tocopherol against flubendiamide or copper-induced micronuclei formation revealed marked reduction in micronuclei formation by all four test antioxidants. The order of ameliorative efficacy of these antioxidants on equimolar basis (10 μM) against flubendiamide was resveratrol > curcumin ≈ catechin > α-tocopherol while resveratrol ≈ α-tocopherol > curcumin > catechin against copper-induced micronuclei formation (Table 2).

### DNA fragmentation

**Fig. 4:**
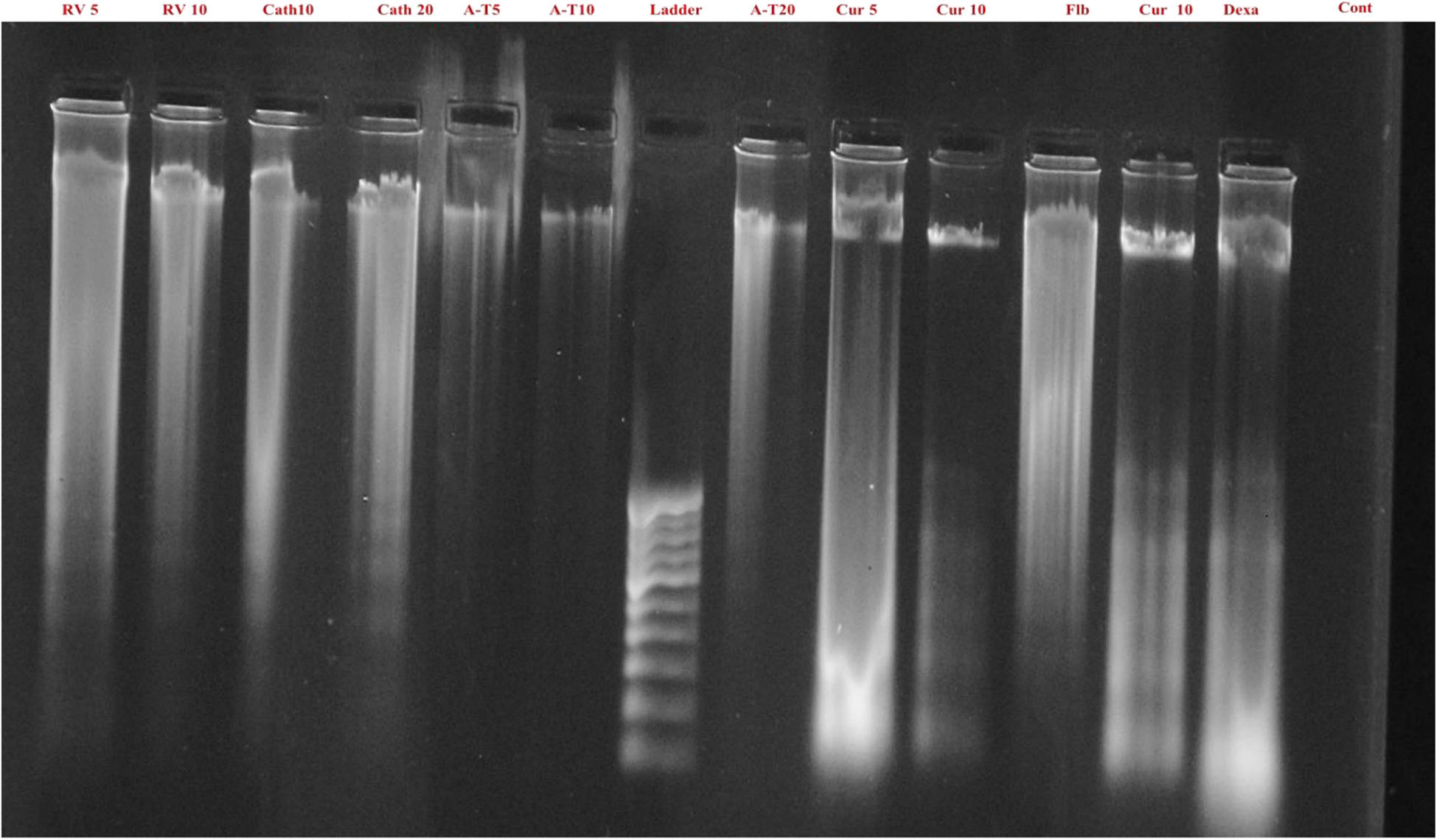
In vitro effect of median lethal concentration of flubendiamide and natural antioxidants at different concentrations on DNA fragmentation pattern in rat splenocytes. RV: Resveratrol (5 and 10 μM), Cath: Catechin (10 and 20 μM), A-T: α-tocopherol (5, 10 and 20 μM), Cur: Curcumin (5 and 10 μM), Flb: Flubendiamide, Dexa: Dexamethasone,Cont: Control

**Fig. 5:**
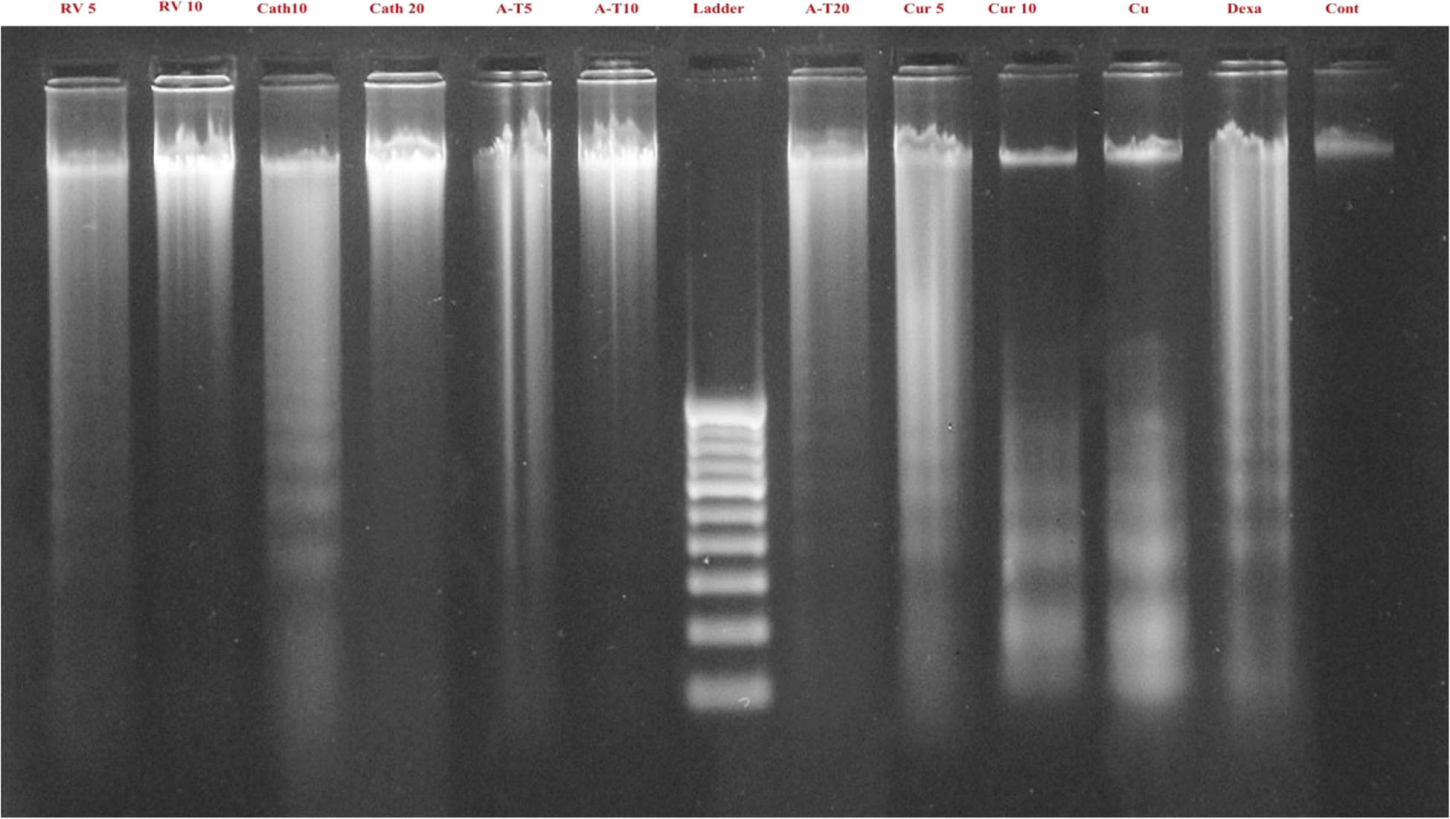
In vitro effect of median lethal concentration of copper and natural antioxidants at different concentrations on DNA fragmentation pattern in rat splenocytes. RV: Resveratrol (5 and 10 μM), Cath: Catechin (10 and 20 μM), A-T: α-tocopherol (5, 10 and 20 μM). Cur: Curcumin (5 and 10 μM), Cu: Copper, Dexa: Dexamethasone, Cont: Control

DNA of the flubendiamide, copper and dexamethasone treated splenocytes showed more shearing compared to the DNA of untreated splenocytes. DNA of the splenocytes treated concurrently with flubendiamide and equimolar concentration (10 μM) of resveratrol, catechin or α-tocopherol also showed almost similar pattern of DNA shearing as observed in the DNA of flubendiamide alone treated splenocytes (Fig. 4). But DNA samples from curcumin (10 μM) + flubendiamide treated splenocytes showed less shearing compared to those treated with resveratrol + flubendiamide, catechin + flubendiamide or α-tocopherol + flubendiamide. Just like flubendiamide and curcumin treated splenocytes, DNA samples from copper + curcumin treated splenocytes also showed comparatively less shearing than in the DNA from splenocytes treated with copper and other antioxidants (resveratrol, catechin, α-tocopherol) as shown in Fig. 5.

### Comet formation

**Fig. 6:**
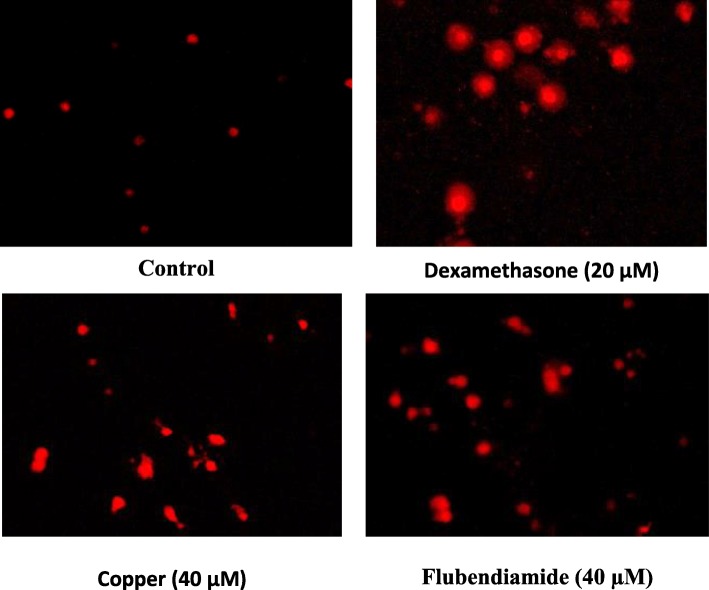
Representative photographs of rat splenocytes showing comet formation following their in vitro exposure to median lethal concentrations of flubendiamide and copper

Comet formation data in splenocytes following their exposure to flubendiamide (40 μM), copper (40 μM) and dexamethasone (20 μM) alone revealed 22.90, 29.59 and 27.69% comets formation compared to 3.09% in negative control and 4.58% in DMSO-treated splenocytes (Table 2; Fig. 6). Resveratrol, catechin, curcumin and α-tocopherol (10 μM each) were found to reduce the percentage of comets formed in flubendiamide and copper-treated splenocytes and the effect of all these agents was concentration-dependent (Table 2). Further, the ameliorative efficacy potential of these antioxidants on equimolar basis against flubendiamide was α-tocopherol ≈ curcumin > catechin > resveratrol while resveratrol > curcumin > catechin > α-tocopherol against copper (Table 2).

### Phase II-in vivo chronic toxicity study

Oxidative stress biomarkers data of rat spleens after 90 days of daily oral exposure to copper and flubendiamide alone and both these in combination (copper + flubendiamide) and those treated simultaneously with α-tocopherol and test xenobiotics are presented in Table 3. Lipid peroxidation levels in rats of the groups exposed to flubendiamide or copper alone and copper + flubendiamide were significantly (*P* < 0.05) higher, while reduced glutathione (GSH) levels in flubendiamide and copper alone and copper + flubendiamide treated groups were moderately decreased when compared with the control group. Similarly, glutathione peroxidase (GPx) activity was found to be significantly (*P* < 0.05) decreased in rat groups exposed to copper, flubendiamide and copper + flubendiamide compared to group I rats. Glutathione-S-transferase (GST) activity in flubendiamide alone group was significantly (*P* < 0.05) lower (0.61 ± 0.12 μM of CDNB-GSH conjugate min-^1^ mg-^1^ protein) than in rest of the groups (Table 3). Further significant (*P* < 0.05) decrease in SOD activity was also observed in copper alone exposed group. Total protein content and catalase activity did not differ significantly between the control and any of the xenobiotics-treated groups. On simultaneous exposure of rats to xenobiotics and α-tocopherol, decrease in lipid peroxidation level and improvement in antioxidants (SOD, GST, GPx, catalase and GSH) cellular defense in splenic tissue of rats were observed compared to the rats exposed to xenobiotics alone.

**Fig. 7:**
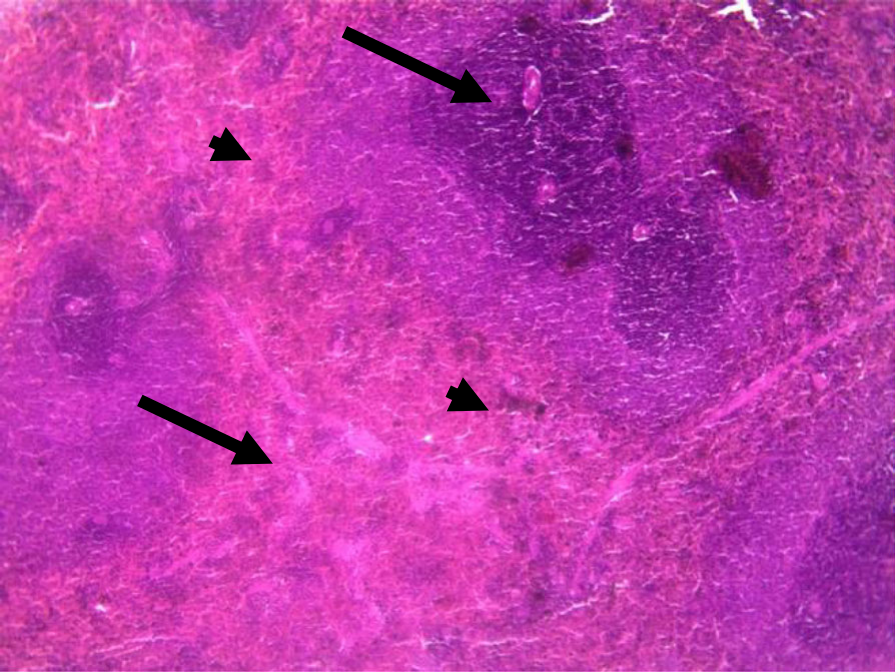
Section of spleen of rat from control group showing healthy histoarchitecture with red (arrow head) and white pulp (arrow) in splenic parenchyma (10X H&E stain)

**Fig. 8:**
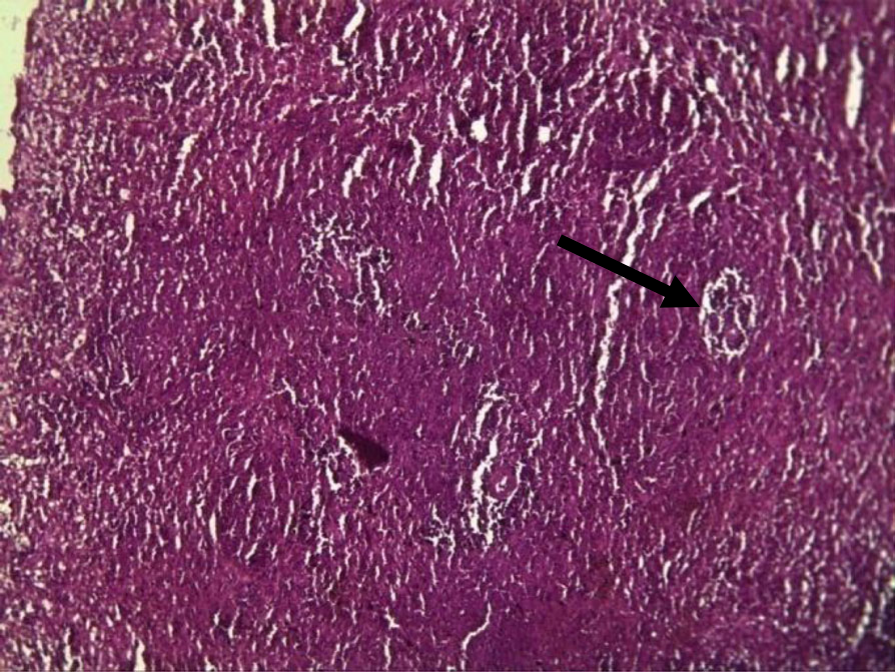
Spleen section of copper sulphate (33 mg/kg) exposed group (IV) showing mild depletion of lymphoid tissue from white pulp (arrow) (10 X H&E stain)

**Fig. 9:**
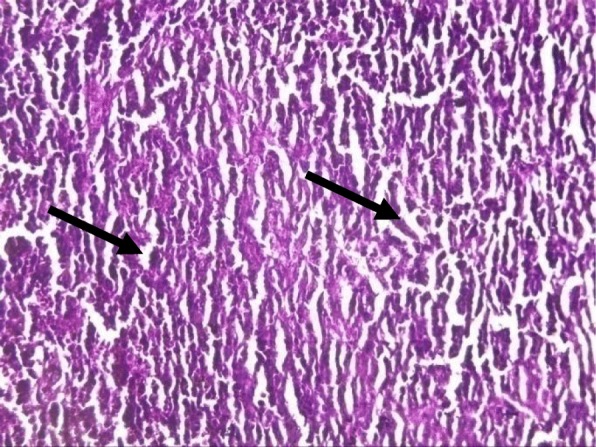
Spleen section of flubendiamide (200 mg/kg) exposed group (V) showing separation of splenocytes and rarefication (arrow) in splenic parenchyma (10 X H&E stain)

**Fig. 10:**
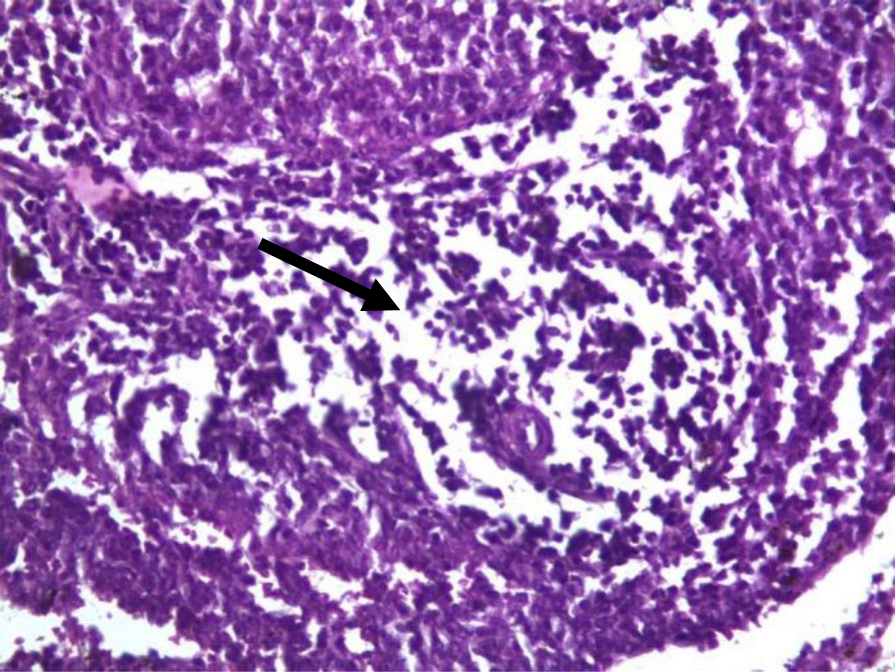
Spleen section of flubendiamide (200 mg/kg) + copper sulphate (33 mg/kg)-exposed group (VI) showing separation of splenocytes and rarefication (arrow) in splenic parenchyma (40 X H&E stain)

**Fig. 11:**
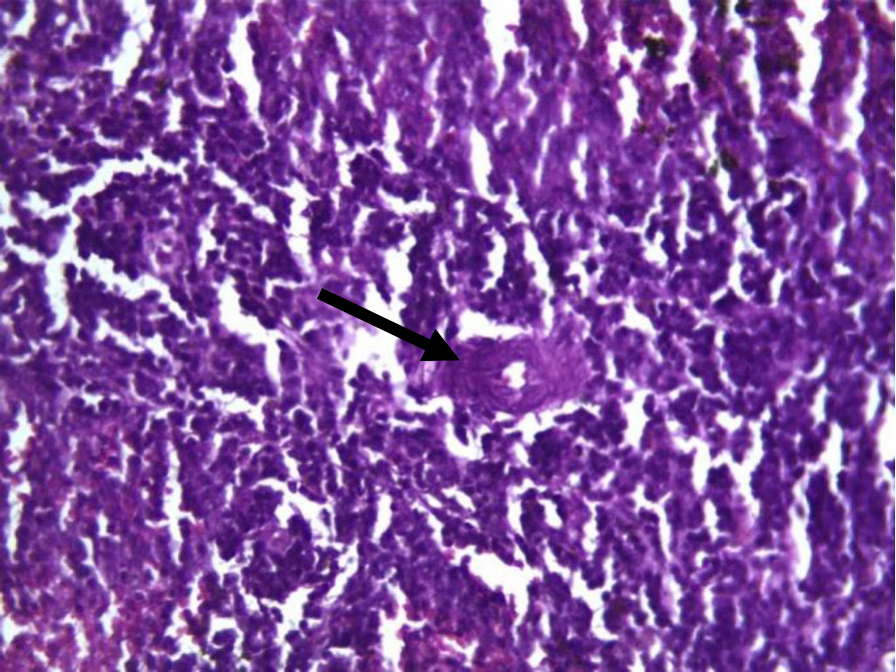
Spleen section of rats treated with α-tocopherol (100 mg/kg) + copper sulphate(33 mg/kg) of group (VII) showing normal histo-architecture with abundant lymphoid tissue in the white pulp (arrow) suggestive of amelioration (40 X H&E stain)

**Fig. 12:**
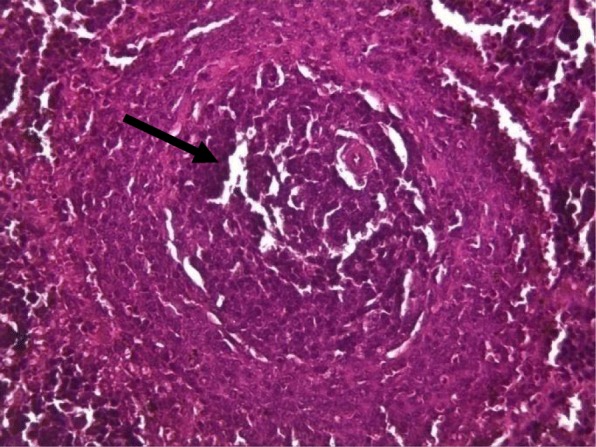
Spleen section of rats treated with α-tocopherol (100 mg/kg) + flubendiamide (200 mg/kg) of group (VIII) showing normal histoarchitecture, abundant lymphoid tissue in the white pulp (arrow) suggestive of amelioration (40 X H&E stain)

**Fig. 13:**
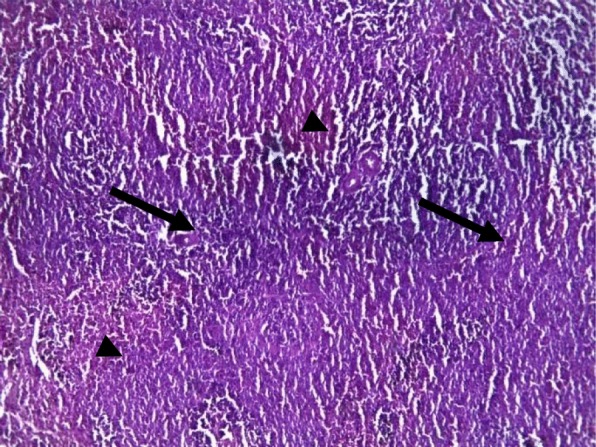
Spleen of rats treated with α-tocopherol (100 mg/kg) + flubendiamide (200 mg/kg) + copper sulphate (33 mg/kg) of group (IX) showing apparently healthy histoarchitecture with ample red (arrow head) and white pulp (arrow) in splenic parenchyma suggestive of amelioration (10 X H&E stain)

Rat spleens from the control groups (I, II and III) exhibited normal histoarchitecture characterized by normal red and white pulps (Fig. 7). Spleen sections of copper sulphate group (IV) rats showed mild depletion of the lymphoid tissue from the white pulp (Fig. 8). Flubendiamide alone (V) group spleen showed separation of splenocytes and rearification in splenic parenchyma (Fig. 9). But spleen sections of copper + flubendiamide treatment group (VI) exhibited separation of splenocytes and rearification in splenic parenchyma (Fig. 10). Concurrent treatment of the rats of groups VII, VIII and IX with α-tocopherol and copper sulphate, α-tocopherol and flubendiamide, and α-tocopherol and combination of flubendiamide and copper, respectively, showed almost normal histoarchitecture of spleens as shown in Figs. 11, 12 and 13, respectively.

## Discussion

Humans and animals are being continuously exposed to mixture of agrochemicals and metals due to agricultural practices, working in heavy metals infested environment and use of ectoparasiticides and other chemicals in household practices [50, 51]. Therefore, an interaction study between different xenobiotics in human and animal systems due to concurrent exposure to these chemical moieties along with their remedial measures seems very important.

No information is available on the cyto-genotoxic effects of flubendiamide and its possible mechanism. Markedly higher percentage of nonviable splenocytes in flubendiamide and copper treated groups evidently suggests cytotoxic effects of flubendiamide and copper in rat splenocytes similar to those reported with certain neonicotinoid insecticides in human peripheral blood lymphocytes [52]. Increase in the number of Tunel+ve cells in the present study are in agreement with increase in number of Tunel+ve germ cells in seminiferous tubules of imidacloprid-treated rats [53] and Tunel+ve fragmented DNA in brain and hippocampus of copper-treated mice and rats [54, 55]. Our findings suggest the ability of flubendiamide and copper to interact with double-stranded DNA (dsDNA) and induce cellular damage which enables TdT to bind with 3’OH label blunt ends of dsDNA and serve as a marker of apoptosis.

Micronuclei assay is one of the most sensitive DNA damage indicator tests and is widely used for evaluation of genotoxic potential of environmental contaminants [56]. Micronuclei assay is employed to detect clastogen and aneugen properties of xenobiotics and determine mitotic delay, apoptosis, chromosome breakage, chromosome loss and non-disjunction potential of xenobiotics [57, 58]. Increase in frequency of micronuclei formation in flubendiamide-treated splenocytes was almost comparable to that induced by dexamethasone. Similar micronuclei forming effect of chlorpyrifos in fish erythrocytes [59] and imidacloprid in human peripheral blood lymphocytes has been reported [60]. Copper-induced increase in frequency of micronuclei formation in rat splenocytes is also in agreement with the observations in bone marrow cells of mice following exposure to copper, and erythrocytes [61, 62], gill and liver cells of fish following exposure to cadmium and copper [63]. Other genotoxicity studies have also suggested that copper is a clastogenic agent [62, 64].

Apart from increase in number of Tunel+ cells and micronuclei formation, DNA fragmentation and comet assay studies also revealed that interaction of flubendiamide or copper with splenocyte cells resulted in DNA damage which is manifested in the form of DNA strand breaks, laddering appearance of DNA in electrophoretic field and comet formation in alkaline conditions. Comet may be formed due to DNA single strand breaks, DNA double strand breaks, DNA adduct formations, DNA-DNA and DNA-proteincross-links or due to interaction of these xenobiotics with DNA [65, 66]. Similar DNA fragmentation in human peripheral blood mononuclear cells [67] and DNA fragmentation along with decrease in cell viability in HepG2 cells following exposure to copper has also been documented [68].

Superoxide dismutase, catalase and glutathione peroxidase are the main defense against free radicals-induced oxidative stress and these act in concert with reduced glutathione and other antioxidants such as α-tocopherol and selenium that protect against the adverse effects of ROS [69]. Increased lipid peroxidation, a decrease in activities of antioxidant enzymes (SOD, GST, GPx) and GSH, separation of splenocytes, and rearification of splenic parenchyma revealed cellular damaging effects of flubendiamide and copper due to generation of oxidative stress. Depletion of GSH occurs as a result of excessive GSH consumption during oxidative stress [70, 71]. Further, GSH is not only a substrate for GPx, but is also involved in electrophile detoxification, free radical scavenging, α-tocopherol generation, phase II conjugation and other reactions [72, 73]. Glutathione-S-transferase catalyzes conjugation of glutathione with a number of electrophilic xenobiotics and prevents their interaction with cellular proteins and nucleic acids, and plays an important role in cellular defense against these xenobiotics [74, 75]. Therefore, inadequate detoxification of flubendiamide or copper or both these in combination, which amplified ROS generation and resulted in oxidative damage, may be responsible for these test compounds-induced decrease in membrane potential and increase in permeability to H^+^ and other ions, and eventually the cell contents release [76].

Copper-induced cyto-genotoxicity in the present study seems to be due to propensity of free Cu ions to participate in formation of ROS by redox cycling and copper-induced formation of hydroxyl radicals from hydrogen peroxide via Haber-Weiss reaction [77–79]. Lipid peroxy radicals damage cells by changing the fluidity and permeability of cell membrane or by attacking the cellular DNA molecule, leading to DNA strand brakes, oxidation of its bases and other intracellular molecules such as proteins [80, 81]. Copper-induced oxidative stress and apoptosis in kidney via intrinsic and extrinsic apoptotic pathways is also well documented [82].

Simultaneous treatment of splenocytes with flubendiamide or copper and either of these along with resveratrol, curcumin, catechin or α-tocopherol resulted in marked decrease in percentage of non-viable splenocytes, Tunel+ve cells, and micronuclei and comet formation in splenocytes. Thus evidently suggests the ameliorative potential of these natural antioxidants against flubendiamide and copper-induced cytogenotoxic effects. The protective effect of resveratrol against xenobiotics is linked to decrease in intracellular ROS accumulation, reactive oxygen intermediate (ROI) generation and lipid peroxidation [83, 84]. Attenuation of pyrogallol-induced hepatic toxicity and oxidative stress changes in hepatic damage and alterations in xenobiotic metabolizing enzymes by resveratrol has also been reported in Swiss mice [85].

Antiapoptotic property of catechin against copper is linked to chelation of Cu^2+^ and formation of an inactive complex with this metal, and thus prevention of generation of potentially damaging free radicals [86, 87]. Similar antiapoptotic, antioxidant and neuroprotective action of green tea extract, rich in various polyphenols, including catechin, against deltamethrin-induced neurotoxicity by improving oxidative status and DNA fragmentation, and suppressing the expression of apoptotic TP53 and COX2 genes has been reported in male rats [88]. Possibility of involvement of similar protective mechanisms of action of the test antioxidants against copper and flubendiamide-induced cytotoxic effects cannot be ruled out.

Curcumin has been reported to ameliorate the arsenic and fluoride-induced genotoxicity in human peripheral blood lymphocytes [33]. Even curcumin has been demonstrated to be effective against radiations-induced hazards [89]. Protective effect of curcumin against different xenobiotics has been attributed to its ability to decrease ROS generation, apoptosis, DNA fragmentation, and cell cycle arrest [33]. Anti-cytogenotoxic effect of curcumin in the present study against flubendiamide and copper could be attributed to its unique conjugated structure which facilitates the coupling reaction at 3′ position of the curcumin with lipids or due to its typical radical trapping ability as a chain-breaking antioxidant that inhibits lipid peroxidation and reduces oxidative stress [90–93].

α-tocopherol is lipophilic in nature which facilitates its entry through cell membrane and thereby quenches free radical species, terminates lipid peroxidation chain reaction and thus interferes with initiation and progression of xenobiotics-induced oxidative damage [94, 95]. α-tocopherol produced protective effect against flubendiamide and copper-induced oxidative stress in splenic tissues by modulating the oxidant-antioxidant mechanisms as substantiated by the altered values of different oxidative stress biomarkers. It also normalized the spleen histoarchitecture towards almost normal as observed in rats of control groups. This evident ameliorative potential of α-tocopherol is in agreement with our previous findings that studied flubendiamide and copper induced testicular injury [96]. Similar preventive effects of α-tocopherol against copper and cadmium-induced cytotoxicity in COS-7 cells [81] and carbofuran-induced genotoxicity in human lymphocytes has also been reported [97].

## Conclusions

Summing up the findings of our in vitro and in vivo studies, it is apparent that flubendiamide and copper-induced alterations in oxidative stress biomarkers interact with cellular subcomponents, especially DNA and result in cytotoxic-insult to splenocytes and spleen-histoarchitecture. Resveratrol is most effective against flubendiamide and curcumin against copper-induced cytotoxic effects, therefore, both these natural phyotconstituent antioxidants hold promising potential for their use in fortifying the conventional food-ingredients to prevent the adverse effects of xenobiotics on human and animals health. However, further studies on signaling intermediary steps and alterations in gene-expression are also warranted.

## Data Availability

Data-sets generated and/or analyzed during the current study are available in the thesis submitted by the first author in the University library and also available with the corresponding author on reasonable request.

## Abbreviations

LPO: Lipid peroxidation
GSH: Reduced glutathione
CAT: Catalase
GPx: Glutathione peroxidase
GST: Glutathione-S-transferase
SOD: Superoxide dismutase
ROS: Reactive oxygen species
IAEC: Institutional Animals Ethics Committee
PBS: Phosphate buffer saline
RPMI: Roswell Park Memorial Institute
HBSS: Hank’s balanced salt solution
SDS: Sodium dodecyl sulfate
NMA: Normal melting agarose
LMPA: Low melting point agarose
DMSO: Dimethyl sulfoxide
